# LONG-TERM EFFECTS OF CHILDHOOD SEXUAL ASSAULT AMONG OLDER PEOPLE WITH HIV

**DOI:** 10.1093/geroni/igz038.2022

**Published:** 2019-11-08

**Authors:** Mark Brennan-Ing, Liz Seidel, Rebecca Erenrich, Stephen E Karpiak

**Affiliations:** 1 Brookdale Center for Healthy Aging, Hunter College, The City University of New York, New York, New York, United States; 2 ACRIA Centers at GMHC, new York, New York, United States; 3 ACRIA Centers at GMHC, San Francisco, California, United States

## Abstract

Research finds high rates of childhood sexual assault (CSA) among people with HIV (PWH). CSA is related to depression, post-traumatic stress disorder, substance abuse, and poor health. PWH age 50 and older account for the majority of this population in the U.S., but we have little information on the impact of CSA on these older adults. Data were obtained from the San Francisco arm of the Research on Older Adults with HIV 2.0 study (n=197). Fifty percent reported CSA. Cisgender women and transgender people were more likely to report CSA compared to other groups. PWH reporting CSA were more likely to meet the diagnostic criteria for PTSD (42% vs. 27%), and had higher mean PHQ-9 depression scores (9.3 vs. 6.8). Those reporting CSA had significantly more comorbid health conditions compared to their peers. Implications for using a trauma-informed care model with older adults living with HIV will be discussed.

